# Prostate epithelial cell lines form spheroids with evidence of glandular differentiation in three-dimensional Matrigel cultures

**DOI:** 10.1054/bjoc.2001.1967

**Published:** 2001-08

**Authors:** S H Lang, R M Sharrard, M Stark, J M Villette, N J Maitland

**Affiliations:** YCR Cancer Research Unit; Department of Biology, University of York, Heslington, York, YO10 5YW, UK; Urogene SA Evry, France

**Keywords:** Matrigel, differentiation, prostate spheroids

## Abstract

Normal (PNT2-C2) and metastatic (PC-3) prostate cell lines were grown in Matrigel to observe the effects on morphology and phenotype in comparison to monolayer culture. In monolayer cultures, PNT2-C2 showed typical round/cuboidal epithelial morphology, with tight cell associations, whereas in Matrigel they formed smooth spheroids, tightly packed with cells. In both monolayer and Matrigel, PNT2-C2 had a differentiated luminal epithelial phenotype with high expression of cytokeratin 8, prostate specific antigen (PSA), prostate specific membrane antigen (PSMA), E-cadherin and desmoglein. In contrast, PC-3 cells possessed an epithelial/mesenchyme morphology in monolayer with loose cell to cell contact and pseudopodial extensions. Immunohistochemical phenotyping indicated the cells were undifferentiated, expressing high levels of vimentin, β1 integrin, CD44 and low expression of cytokeratin 8. In Matrigel they formed smooth and irregular spheroids, which had a lumen surrounded by a single cell layer. Matrigel also influenced the expression of PSA, PSMA and CD44. These results indicate that Matrigel culture can induce morphological differentiation of prostate cancer cells which initially had a basal phenotype. © 2001 CancerResearch Campaign http://www.bjcancer.com


*British Journal of Cancer* (2001) **85**(4), 590–599
© 2001 Cancer Research Campaign
doi: 10.1054/ bjoc.2001.1967, available online at http://www.idealibrary.com on IDEAL

http://www.bjcancer.com

### Prostate epithelial cell lines form spheroids with evidence of glandular differentiation in three-dimensional Matrigel cultures

**SH Lang[1], RM Sharrard[1], M Stark[2], JM Villette[3] and NJ Maitland[1]**

[1]YCR Cancer Research Unit, [2]Department of Biology, University of York, Heslington, York YO10 5YW, UK; and [3]Urogene SA, Evry, France

**Summary** Normal (PNT2-C2) and metastatic (PC-3) prostate cell lines were grown in Matrigel to observe the effects on morphology and phenotype in comparison to monolayer culture. In monolayer cultures, PNT2-C2 showed typical round/cuboidal epithelial morphology, with tight cell associations, whereas in Matrigel they formed smooth spheroids, tightly packed with cells. In both monolayer and Matrigel, PNT2-C2 had a differentiated luminal epithelial phenotype with high expression of cytokeratin 8, prostate specific antigen (PSA), prostate specific membrane antigen (PSMA), E-cadherin and desmoglein. In contrast, PC-3 cells possessed an epithelial/mesenchyme morphology in monolayer with loose cell to cell contact and pseudopodial extensions. Immunohistochemical phenotyping indicated the cells were undifferentiated, expressing high levels of vimentin, β1 integrin, CD44 and low expression of cytokeratin 8. In Matrigel they formed smooth and irregular spheroids, which had a lumen surrounded by a single cell layer. Matrigel also influenced the expression of PSA, PSMA and CD44. These results indicate that Matrigel culture can induce morphological differentiation of prostate cancer cells which initially had a basal phenotype. © 2001 Cancer Research Campaign http://www.bjcancer.com

**Keywords**: Matrigel; differentiation; prostate spheroids

Normal growth and morphology of epithelium is essential in the establishment of a developing and functioning organ. Such processes are driven by an epithelial cell's genotype but also by its interaction with surrounding stroma and basement membranes. Production of the basement membrane is an active process, governed by both the epithelium and the stroma in the tissue (Streuli and Bissel, 1990; Smola et al, 1998).

The commercially available basement membrane preparation, Matrigel, has been widely used to study epithelial interactions with the basement membrane. Matrigel is composed primarily of laminin and collagen IV, but includes other matrix proteins, and several growth factors and proteases. Studies, particularly in breast, have revealed that the maintenance of cell morphology and expression of tissue specific genes are both intimately related to the expression of basement membrane. Breast epithelial cells cultured in Matrigel gave rise to three dimensional acinar structures capable of secreting milk proteins in a luminal direction (Chen and Bissell, 1989; Barcellos-Hoff et al, 1989). Similar work with prostate epithelia has also shown the formation of acini capable of secreting prostate specific proteins (Webber et al, 1997). Studies with the prostate have further shown that stromal cells are also required for growth and functional differentiation of prostate epithelia, though the contribution of basement membranes was not established (Timms et al, 1995; Liu et al, 1997; Bayne et al, 1998).

The progression of prostate adenocarcinoma is accompanied by the loss of normal epithelial ductal architecture and the breakdown or altered synthesis of basement membrane (Knox et al, 1994; Nagle et al, 1995). Progression is also accompanied by altered cell adhesion molecule expression, such as integrin receptors for matrix proteins, and cadherins, and aberrant communication between epithelia and stroma. This leads to the loss of the correct instructions for basement membrane formation and undoubtedly contributes to the loss of normal epithelial morphology and differentiation, accelerating the emergence of a tumour cell (Weaver et al, 1996).

The aim of our experiments was to compare the morphology and phenotype of epithelia from normal and tumour tissue grown on a basement membrane capable of inducing normal epithelial differentiation and morphology (Matrigel). We have previously characterized several prostate cell lines on the basis of their in vitro metastatic qualities and response to stromal cultures (Lang et al, 2000). The prostate epithelial cell line PNT2-C2 (immortalized normal epithelium) was identified as the least invasive and non-motile, whilst PC-3 (metastatic) was found to be both highly invasive and motile. These two cell lines were used to compare cellular morphology (by phase and electron microscopy) and phenotype (immunohistochemistry and reverse transcriptase-polymerase chain reaction) in Matrigel, to cells grown in normal tissue culture monolayer.

### MATERIALS AND METHODS

### Materials

General chemicals were purchased from Sigma (Poole, UK) unless stated and tissue culture plastic from (Corning Costar Ltd, High Wycombe, UK). Antibodies were purchased from Dako (High Wycombe, UK) unless stated.

*Received 14 November 2000*
*Revised 14 May 2001*
*Accepted 17 May 2001*

*Correspondence to:* SH Lang

### Cell line culture

PNT2-C2 is an immortalised normal human prostate epithelial cell line (Cussenot et al, 1991; Berthon et al, 1995). It was routinely cultured in RPMI 1640 culture media (Life Technologies, Paisley, UK) supplemented with 10% fetal calf serum (PAA Laboratories, GmbH, Linz, Austria) and 2 mM glutamine (Life Technologies).

PC-3 and LNCaP are human prostate adenocarcinoma cell lines, derived from a bone metastasis and lymph node respectively, both were obtained from the European Collection of Animal Cell Cultures (Porton Down, UK). PC-3 was routinely cultured in Ham's F12 culture media (ICN, Basingstoke, UK) supplemented with 7% fetal calf serum and 2 mM glutamine, whilst LNCaP were cultured with the same media for PNT2-C2 cells. All cell lines were routinely cultured without antibiotics in a humidified atmosphere at 37˚C and 5% $CO_2$.

### Cell line culture in Matrigel

Cells were prepared in their normal growth media to a concentration of 60 000 cells/ml. On ice they were mixed 1:1 (*v/v*) with Matrigel (Becton Dickinson, Oxford, UK) and 0.5 ml aliquots were subsequently plated into 24-well plates. The Matrigel was set by incubating at 37˚C for 30 min, after which 1 ml of normal growth media was added. Cells were thereafter medium changed every 3 days, by the removal of 0.5 ml of spent media and the addition of 0.5 ml of fresh media.

### Electron microscopy

Cells growing in monolayer were prepared in 25 ml tissue culture flasks. Cells growing in matrigel were prepared in 24 well plates. Both cell preparations were washed twice with phosphate buffered saline (PBS) and then fixed for 1 h at room temperature in 100 mM phosphate buffer, 4% paraformaldehyde (TAAB, UK) and 2.5% Ultrapure glutaraldehyde. Cells were posted fixed with 1% $OsO_4$ for 1 h on ice, washed and dehydrated through ascending concentrations of alcohol.

For transmission electron microscopy (TEM), infiltration was carried out using ethanol/propylene oxide (1:1) for 1 h. Specimens were then placed in fresh resin and polymerized at 60˚C for 48 h. Thick sections were cut at 1 µm and stained with 0.6% toluidene blue in 0.3% sodium bicarbonate. 70 nm sections were cut and stained with saturated uranyl acetate in 50% ethanol followed by Reynolds lead citrate and observed with a Jeol JEM 1200 Ex transmission electron microscope.

For scanning electron microscopy, specimens were air dried from hexamethyldisilazane in a desiccator overnight. They were then sputter coated with gold using a Polaron E5000 coater and observed using a Hitachi S-2400 scanning electron microscope.

### Fluorescent immunostaining

To analyse cells grown in monolayer, cells were plated in glass chamber slides (Nunc, Illinois, USA), in their normal growth media and cultured until approximately 70% confluent. They were then washed twice with PBS and fixed for 10 min in 4% paraformaldehyde followed by 10 min in 70% ethanol.

Cells grown in Matrigel were snap frozen in liquid nitrogen after embedding the gel in OCT Compound (BDH, Poole, UK). Embedded gels were stored at –20˚C. 10 µm sections were cut on a cryostat and mounted onto Super frost microscope slides (BDH).

Immunostaining was carried out according to Table 1. Antibodies were prepared in PBS supplemented with 1% bovine serum albumin. Each step was followed by three washes in PBS. Primary antibodies were incubated at room temperature for 1 h and secondary antibodies for 30 min. Spheroids were counter stained with 1 µg/ml 4′, 6-diamidino-2-phenylindole (DAPI). Coverslips were mounted to slides using Citifluor (Agar Scientific Limited, Stansted, UK). Immunostained cultures were observed using a Nikon Eclipse TE300 fluorescent microscope.

### Reverse transcriptase-PCR (RT-PCR)

RNA was prepared from PC3 and LNCaP cells using RNAzol B essentially according to the method of Chomczynski and Sacchi (1987). 10 µg of total RNA from each cell line was primed with 0.25 µg oligo (dT) 12–15 (Life Technologies) and reverse-transcribed to cDNA in a 20 µl reaction containing 50 mM tris HCl pH 8.3, 75 mM KCl, 3 mM $MgCl_2$, 10 mM dithiothreitol, 1 mM dNTPs and 100 units Superscript II reverse transcriptase (Life Technologies), incubated at 45˚C for 120 min. Equal volumes of each of the cDNA preparations were PCR-amplified in 20 µl reaction mixture containing 200 µM dNTPs, 1.5 mM $MgCl_2$, 0.7 µM of each primer, 1 × Expand buffer and 1 unit of Expand High Fidelity DNA polymerase (Boehringer), using the primers 5′-GGGGGCCCACTTGTCTGTAAT-3′ and 5′-ATCCCACCCCT-CTTTCATCTCTG-3′, which amplify a 450 bp segment of the

**Table 1** Antibodies and immunostaining procedures

| Antigen | Clone/sera | Antibody supplier | Dilution | Secondary procedures |
|---|---|---|---|---|
| Cytokeratin 8 | M20 | Sigma | 1:100 | RF |
| Cytokeratin 1, 5, 10, 14 | 34βE12 | Dako | 1:50 | RB, SF |
| Vimentin | VIM 13.2 | Sigma | 1:200 | RF |
| PSA | rabbit | Dako | 1:20 | SB, SF |
| PSMA | PSM-P12 | JM Villete, St. Louis Hospital, Paris | 1:100 | RF |
| Desmoglein | CBL 174 | Cymbus Biotechnology | 1:10 | RB, SF |
| E-Cadherin | HECD-1 | R&D | 10 µg/ml | RB, SF |
| β1 integrin | sc-9970 | Santa Cruz | 2 µg/ml | RF |
| CD44 | F10 44–2 | Novocastra | 1:10 | RB, SF |
| hmet | sc-10 | Santa Cruz | 5 µg/ml | SB, SF |

RF, 1:30 dilution of FITC conjugated rabbit anti mouse; RB, 1:300 dilution of biotinylated rabbit anti-mouse; SF, 1:50 dilution of FITC conjugated streptavidin; SB, 1:300 dilution of biotinylated swine anti-rabbit.

© 2001 Cancer Research Campaign

PSA cDNA spanning the termination codon. Amplification was carried out in a Perkin-Elmer 2400 thermal cycler according to the protocol: 2 min at 94˚C; 30 cycles of 15 s 94˚C, 20 s at 55˚C and 75 s + 2 s/cycle at 72˚C; 5 min at 72˚C. PCR products were analysed by agarose gel electrophoresis. In order to confirm the quality of RNA used and to test the efficiency of the cDNA synthesis reactions, equal volumes of each of the cDNA preparations were also PCR-amplified for 16 cycles using the primers 5′-AAGGTGAAGGTCGGAGTCAA-3′ and 5′-GGA-CACGGAAGGCCATGCCA-3′, which amplify a 700 bp product from the cDNA of the GAPDH gene. Gel analysis confirmed that equal amounts of GAPDH product were amplified from each cDNA sample.

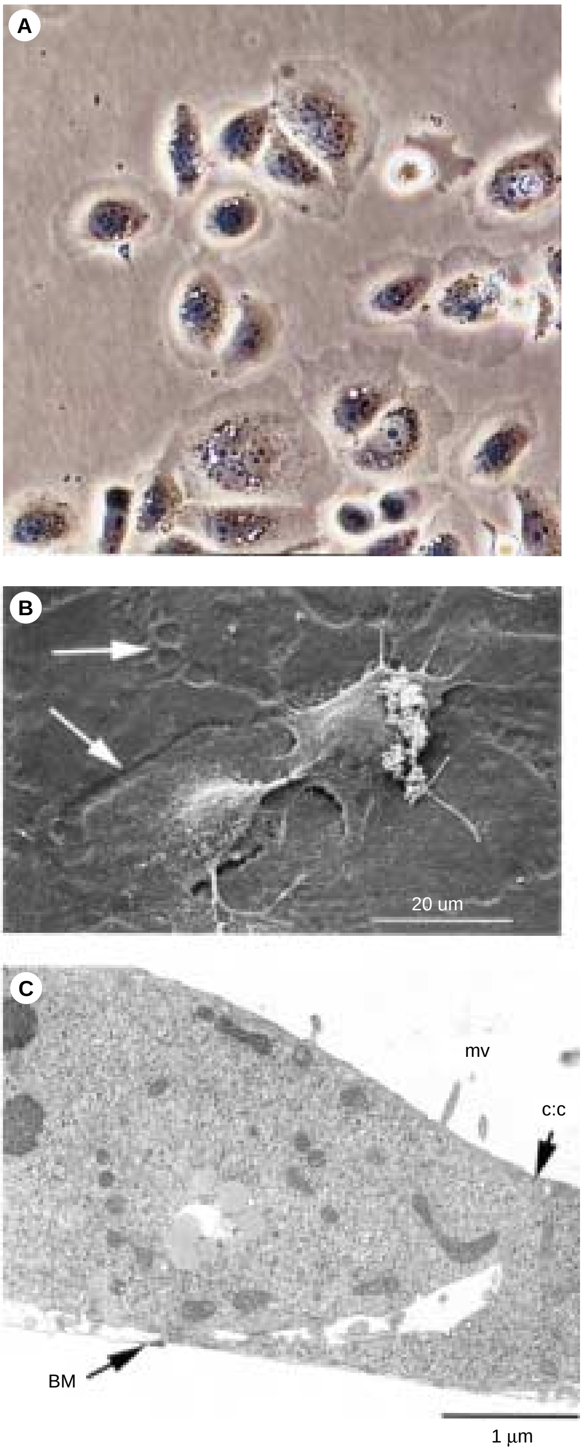

**Figure 1** Morphology of PNT2-C2 in monolayer culture. (**A**) Phase contrast microscopy of a sub-confluent culture. (**B**) Scanning electron microscopy (arrows indicate the position of cell to cell contacts). (**C**) Transmission electron microscopy of a typical cell shown in contact with another cell. BM, basement membrane; mv, microvilli; c:c, cell to cell contact

### RESULTS

### Morphology of PC-3 and PNT2-C2 in monolayer

PNT2-C2 cells showed a typical epithelial morphology by phase contrast microscopy (Figure 1A). The cells were regular in shape with a rounded morphology which became cuboidal with tight cell to cell associations when confluent. Tight cell to cell contact was further demonstrated by scanning electron microscopy (Figure 1B), which showed that a confluent cell layer was very smooth and that cell junctions were difficult to discern (arrows). Individual cells were difficult to see unless dividing, demonstrated by the two cells in the centre. Transmission electron microscopy (Figure 1C) indicated the cells had a flattened appearance. Analysis of several cells revealed the presence of Golgi bodies and numerous mitochondria, coated pits, rough endoplasmic reticulum and secretory vesicles. The cells showed a small degree of polarity, since they produced a basement membrane and had a few microvilli on the 'apical' surface. The cell to cell contact visible in Figure 1C shows how the tight seal gives a very smooth appearance to the 'apical' surface. Both tight junction and desmosome-like complexes were observed by electron microscopy.

In contrast, PC-3 cells showed a more fibroblast-like morphology in monolayer with very loose cell to cell contacts (Figure 2A). Cells predominantly had pseudopodia-like extensions (some very long) whilst others were round in shape. Time lapse microscopy has shown that cells can exist in either state (results not shown). Scanning electron microscopy (Figure 2B) showed that individual cells were easily discernible and had a more three-dimensional appearance than PNT2-C2. The cells had numerous and extensive microvilli and cell projections, which appeared to attach to other cells. Transmission electron microscopy indicated the cells were rounded in shape and appeared disorganized, especially the microvilli, which were very extensive. Mitochondria, coated pits and secretory vesicles were numerous, whilst Golgi were small. No tight or desmosome-like junctions were observed. The cells showed weak polarity since they also secrete a basement membrane and have numerous microvilli on their 'apical' surface (a typical cell is shown in Figure 2C).

### Phenotype of PNT2-C2 and PC-3 in monolayer

The phenotype of prostate cell lines was identified by immunostaining for a variety of differentiation markers. We looked for the prostate epithelial basal cell markers; basal cytokeratin (1,5,10,14), CD44 and β1 integrin (Robinson et al, 1998; Paradis et al, 1998; Knox et al, 1994) and luminal markers; cytokeratin 8, prostate specific antigen (PSA) and prostate specific membrane antigen (PSMA) (Robinson et al, 1998; Chang et al, 1999). Prostate specific antigen (PSA) and prostate specific membrane antigen (PSMA) also served as functional differentiation markers. Finally, as markers of de-differentiation from the normal epithelial phenotype we analysed vimentin and h-met. In addition the cell

 *© 2001 Cancer Research Campaign*

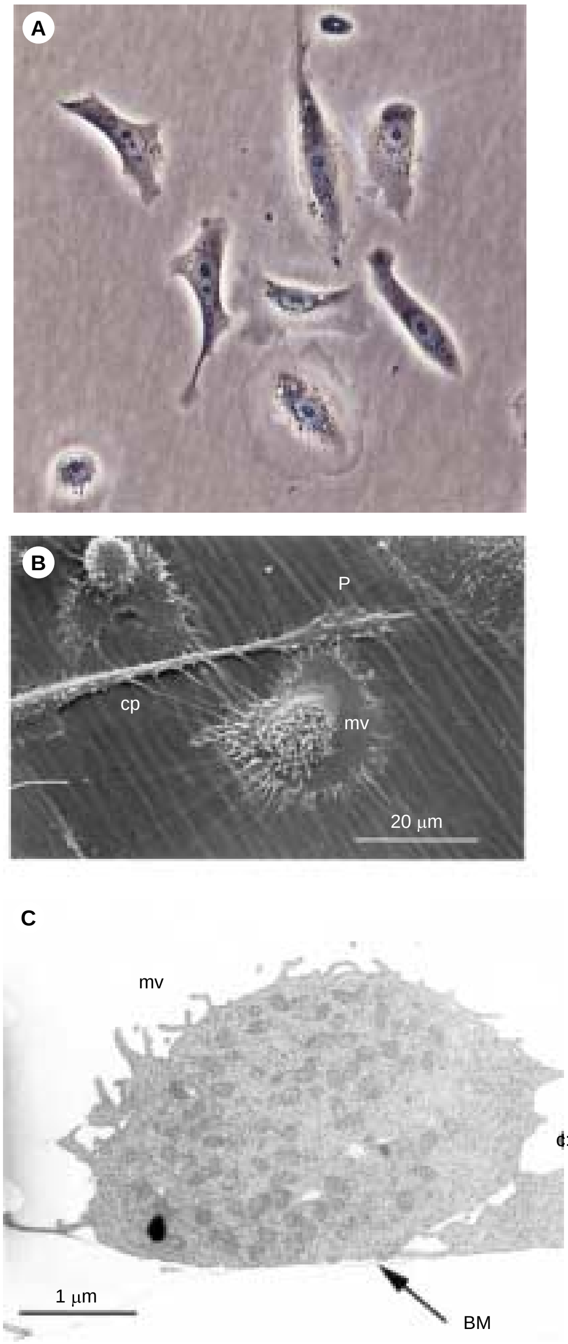

**Figure 2** Morphology of a sub-confluent PC-3 monolayer culture. (**A**) Phase contrast microscopy. (**B**) Scanning electron microscopy. P, pseudopodia; cp, cell projection; mv, microvilli. (**C**) Transmission electron microscopy of a typical cell shown in contact with another. BM, basement membrane; mv, microvilli; c:c, cell to cell contact adhesion molecules, E-cadherin and desmoglein were investigated. The results are summarized in Table 2.

PNT2-C2 epithelia showed high expression of cytokeratin 8 (luminal) and weak staining of cytokeratin 1,5,10,14 (basal), whilst vimentin expression was negligible. PC-3 cells showed almost the opposite expression of high vimentin staining and low expression of both cytokeratins, in all instances the staining was pan-cellular. PNT2-C2 cells showed cytoplasmic expression of prostate specific proteins (PSA and PSMA) and cell membrane expression of cell adhesion molecules (E-cadherin and desmoglein), however PC-3 showed no expression of these proteins. PC-3 did stain strongly for the basal markers, β1 integrin, CD44 and also h-met whilst PNT2-C2 showed only weak staining. β1 integrin (Figure 3) and CD44 expression was localized to the membrane whilst h-met was pan-cellular. A comparison of cytokeratin 8 and β1 integrin immunostaining between PNT2-C2 and PC-3 cells is shown in Figure 3.

### Morphology of PNT2-C2 and PC-3 in Matrigel

After 3 days growth small spheroids were visible in both PNT2-C2 and PC-3 Matrigel cultures. These cultures were grown 7 or 10 days for morphological and phenotypical analysis. In Matrigel, PNT2-C2 cells formed smooth spheroids; their appearance in both phase and scanning electron microscopy was dense and individual cells were difficult to visualize, as in monolayer culture (Figures 4A and 4B). Sections of these spheroids showed that they were tightly packed with cells (Figure 4C). Transmission electron microscopy (Figure 5) showed that the cells were not tightly adherent, but that they attached at intermittent points predominantly using desmosome-like junctional complexes (Figure 6), though tight junctions were also visible. Golgi bodies, mitochondria and rough endoplasmic reticulum were easily visible. Mitotic bodies were evident as well as areas of necrosis which produced small lumens, but the lumens were not surrounded by any polarized epithelial cells.

PC-3 cells formed smooth (Figure 7A) and irregular (Figure 7B) spheroids when cultured in Matrigel. Scanning electron microscopy (Figure 7B) showed that individual cells were easily visible. Sectioning the spheroids showed that the spheroids were hollow (Figure 7C), and were predominantly one cell thick (although sometimes two cell layers were observed). Transmission electron microscopy (Figure 8) indicated that the centre of the spheroid contained necrotic cells or cellular debris. The cells themselves showed greater polarity than in monolayer, since secretory vesicles and microvilli were seen towards or on the luminal surface. Cell to cell contacts were also greater and tight junctions were clearly evident by electron microscopy. Well-defined Golgi were not observed, but more abundant rough endoplasmic reticulum was seen than in monolayer culture.

### Phenotype of PNT2-C2 and PC-3 spheroids grown in Matrigel

Overall PNT2-C2 spheroids showed a similar staining pattern in Matrigel (summarized in Table 2) to that in monolayer culture. Intermediate filaments, prostate secretory proteins and cell adhesion molecules all showed the same intensity and localization of staining in Matrigel as on monolayer. An example of intermediate filament staining is shown in Figure 3, cytokeratin 8 staining was strong and pan-cellular in both monolayer and Matrigel for PNT2-C2 cells. CD44 expression also remained constant, whilst β1 integrin and h-met expression showed a slight increase in intensity when grown in Matrigel. β1 integrin and CD44 expression was located at the cell membrane but was predominantly expressed at the basal membrane (at the spheroid-Matrigel interface), shown in Figure 3. PSA and PSMA expression remained cellular, throughout the spheroid.

PC-3 spheroids showed a low expression of basal cytokeratin and luminal cytokeratins, whilst vimentin expression remained

© 2001 Cancer Research Campaign

**Table 2** Immunohistochemical phenotyping of PNT2-C2 and PC-3 cells grown in monolayer and Matrigel

| Marker | Antigen | Normal luminal expression | Normal basal expression | PNT2-C2 monolayer | Matrigel | PC-3 monolayer | Matrigel |
|---|---|---|---|---|---|---|---|
| Basal epithelium | Cytokeratin 1, 5, 10, 14 | – | +++(1) | $+^{pc}$ | $+^{pc}$ | $+^{pc}$ | $++^{pc}$ |
| | CD44 | – | +++(2) | $+^{m}$ | $+^{mb}$ | $+++^{m}$ | $+^{m}$ |
| Luminal epithelium | Cytokeratin 8 | +++(3) | – | $+++^{pc}$ | $+++^{pc}$ | $+^{pc}$ | $+^{pc}$ |
| Functional | PSA | +++(1) | – | $+++^{pc}$ | $+++^{pc}$ | – | $++^{pc}$ |
| differentiation | PSMA | +++(4) | – | $++^{pc}$ | $+++^{pc}$ | – | $++^{pc}$ |
| Adhesion molecules | E-cadherin | +++(5) | | $++^{m}$ | $++^{m}$ | – | – |
| | Desmoglein | | | $++^{m}$ | $++^{m}$ | – | – |
| Differentiation/metastasis | β1 integrin | – | +++(6) | $+^{m}$ | $++^{mb}$ | $+++^{pcm}$ | $+++^{pcm}$ |
| | h-met | | +++(7) | $+^{pc}$ | $++^{pcm}$ | $++^{pc}$ | $++^{pcm}$ |
| | vimentin | +(8) | – | – | $+^{pc}$ | $+++^{pc}$ | $++^{pc}$ |

(1) Robinson et al 1998, (2) Paradis et al 1998, (3) Sherwood et al 1991, (4) Chang et al 1999, (5) Murant et al 1997, (6) Knox et al 1994, (7) Pisters et al 1995, (8) Fraga et al 1998. Abbreviations for localization of staining patterns are as follows: pc, pan-cellular; m, membrane; b, basal membrane (that between spheroid and Matrigel).

relatively strong when grown in Matrigel. Expression of cell adhesion molecules (E-cadherin and desmoglein) remained negative and β1 integrin and h-met retained a similar staining intensity in Matrigel whilst CD44 expression was much reduced. β1 integrin and CD44 expression were again associated with the cell membrane, but were not strongly expressed at the basal membrane, as for PNT2-C2 cells (Figure 3). Interestingly, expression of prostate specific proteins were detected in Matrigel, when they had been negative in monolayer culture (Figure 9).

### RT-PCR analysis of PSA expression by PC-3 cells in monolayer and Matrigel culture

As it is difficult to quantify expression of PSA by immunostaining when analysing different cellular preparations and morphologies, we further investigated PSA expression by semi-quantitative RT-PCR. Figure 10 indicates that growth of PC-3 cells in Matrigel downregulated PSA expression at the transcriptional level to approximately 60% of that in monolayer culture. In comparison, levels of PSA expression in PC-3 cells were much lower in both Matrigel and monolayer than in LNCaP (included as a positive control) cells grown on monolayer.

### DISCUSSION

Basement membranes are an essential contributor to epithelial function and architecture. Together with stromal cells, basement membranes are essential for directing the formation of epithelial three dimensional acini and their functional differentiation (secretion of tissue specific proteins). The experiments presented here indicate that a human prostate normal epithelial cell line

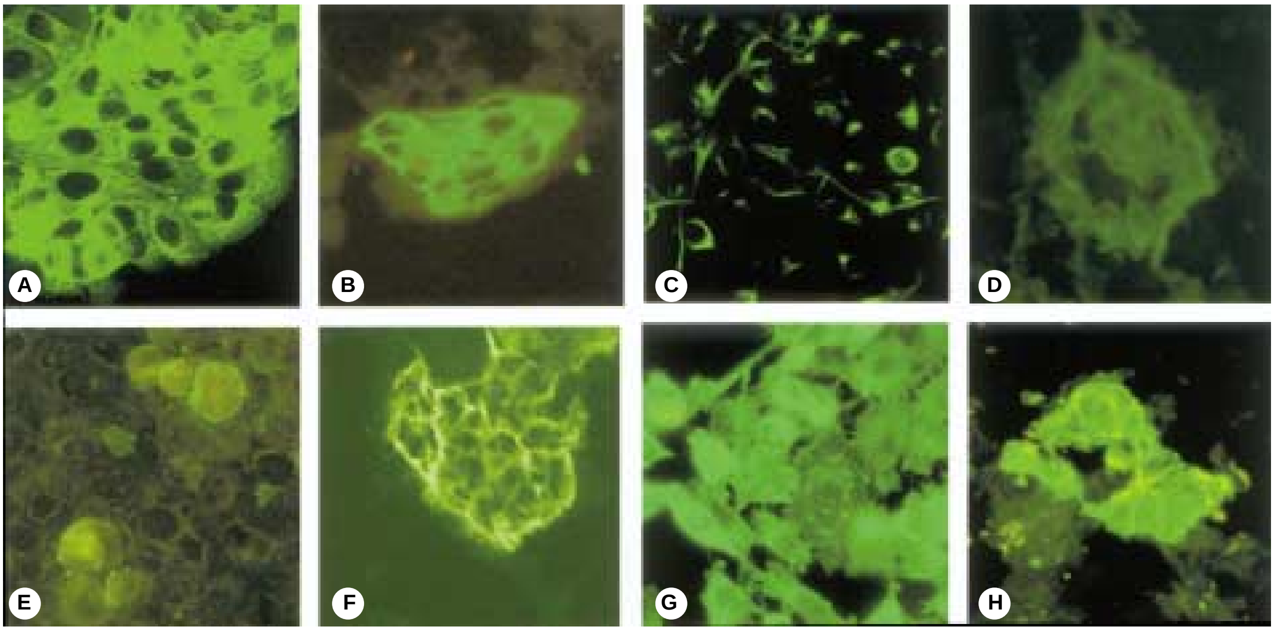

**Figure 3** Immunohistochemical staining of cytokeratin 8 (**A-D**) and β1 integrin (**E-H**) of PNT2-C2 (**A,B,E,F**) and PC-3 cells (**C,D,G,H**) grown in monolayer culture (**A,C,E,G**) or Matrigel (**B,D,F,H**)

 *© 2001 Cancer Research Campaign*

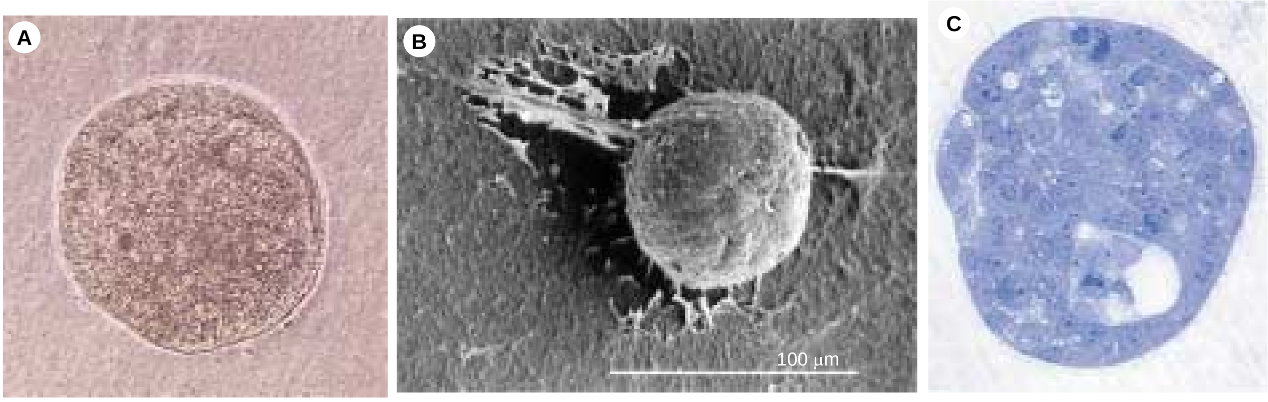

**Figure 4** Morphology of PNT2-C2 spheroids grown in Matrigel. (**A**) Phase contrast microscopy. (**B**) Scanning electron microscopy. (**C**) 1 μm sections stained with toluidene blue

(PNT2-C2) does not form acini which contain either a lumen or polarized epithelium but spheroids of solid cells. Phenotypically these cells showed a differentiated phenotype on monolayer and maintained this in Matrigel. However a metastatic epithelial cell line (PC-3) did form acini with polarized epithelia which expressed PSA and PSMA. These results are inconsistent with those of Webber et al (1997) who found that a different human prostate normal epithelial

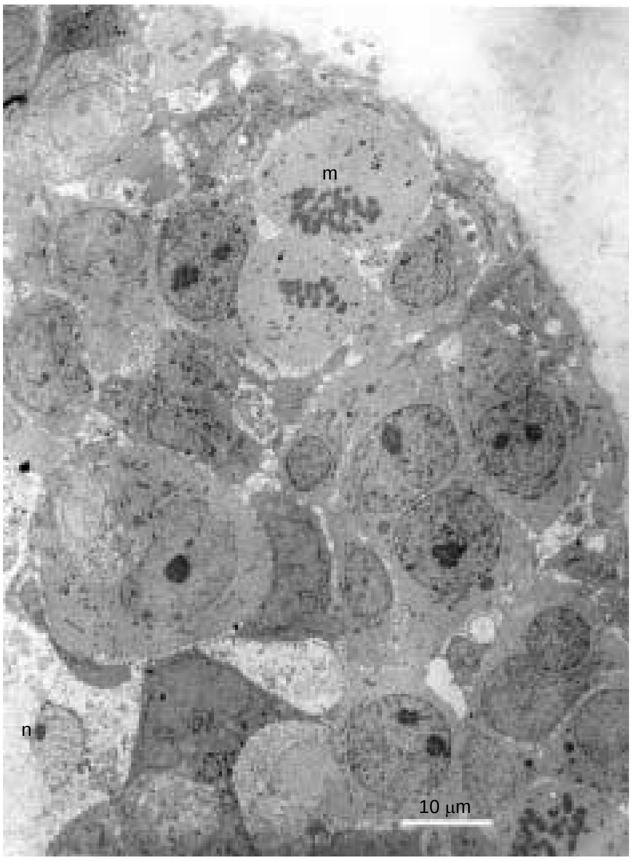

**Figure 5** Transmission electron microscopy of PNT2-C2 spheroids grown in Matrigel. m, mitotic body; n, necrotic lumen

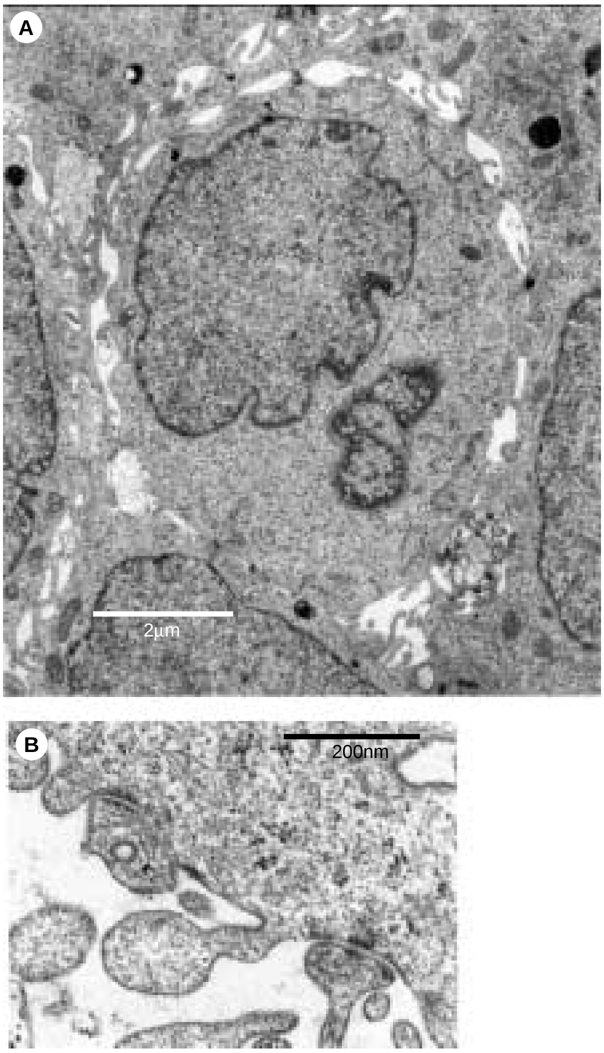

**Figure 6** High magnification transmission electron microscopy of PNT2-C2 spheroids grown in Matrigel, illustrating how cells attach intermittently (**A**) with desmosome-like junctions (**B**)

© 2001 Cancer Research Campaign

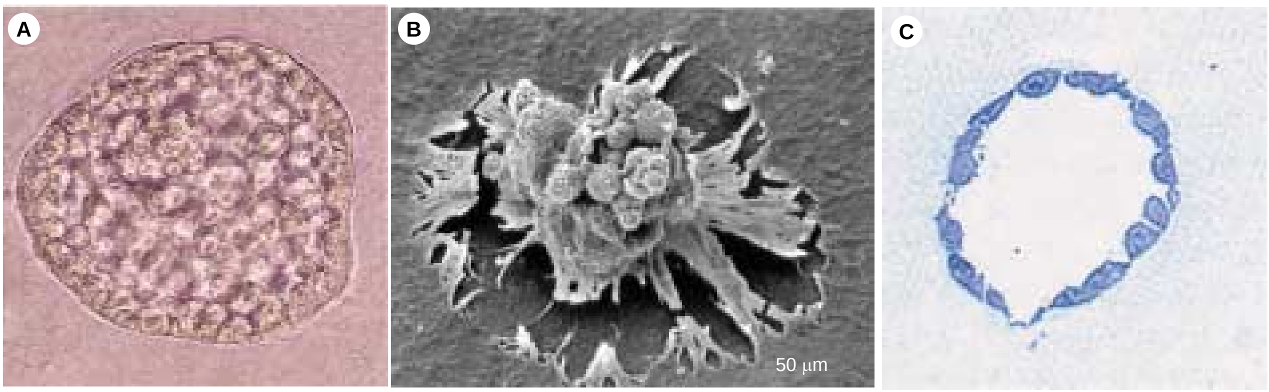

**Figure 7** Morphology of PC-3 spheroids grown in Matrigel. (**A**) Phase contrast microscopy. (**B**) Scanning electron microscopy. (**C**) 1 μm sections stained with toluidene blue cell line (RWPE-1) formed extensive budding and branching tubes. These structures formed acini which had organized and polarized epithelia that secreted PSA. In contrast a different metastatic cell line (DU145) formed amorphous solid balls of cells. In agreement with this latter result, we have also cultured DU145 in Matrigel and found them to form spheroids of solid cells (results not shown). We propose that formation of acinus-like structures in Matrigel is dependent on the differentiation state of a particular epithelial cell type and not its tumorigenic state.

In prostate cell biology is widely believed that basal cells are the precursors of luminal cells and that within the basal cell population is a more primitive stem cell (Robinson et al, 1998; Liu et al, 1997). Phenotypically prostatic luminal epithelium are positive for cytokeratins 8, 18, 19 and PSA and basal epithelium are positive for cytokeratins 10, 11, 14 (Robinson et al, 1998), CD44 (Paradis et al, 1998) and β1 integrin (Knox et al, 1994). Since PC-3 cells show an undifferentiated/basal-like phenotype they may have been able to undergo differentiation in response to the Matrigel, whilst PNT2-C2 cells are already highly differentiated luminal epithelia and therefore could not be induced to undergo further differentiation. The presence of homotypic cell adhesion molecules (E-Cadherin and desmoglein) may result in the formation of large solid balls of 'sticky' cells. The presence of these adhesion molecules undoubtedly accounted for their very close cell junctions seen in scanning microscopy. Most likely if PNT2-C2 cells had not been immortalized with SV40 large T antigen they would undergo terminal differentiation and die. Selection for primary prostatic luminal epithelial cells and subsequent growth in Matrigel in our laboratory has shown that the cells are unable to grow and eventually die (unpublished results). Since Webber et al (1997) did not carry out any phenotyping of their cells, it is difficult to further prove this hypothesis at this time. Further evidence for acini formation in Matrigel requiring an undifferentiated cell comes from our own studies of DU145 cells, which have shown they are more differentiated than PC-3 but are less motile and invasive (Lang et al, 2000). DU145 cells strongly express cytokeratin 8 and E-cadherin (Mitchell et al, 2000) and have only moderate expression of desmoglein and basal cytokeratins, and low expression of CD44 and PSA (manuscript in preparation). This phenotypic profile would be consistent with differentiated cells forming solid spheroids of cells.

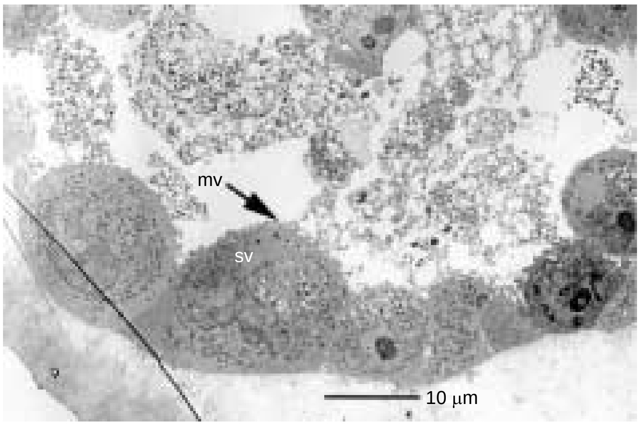

**Figure 8** Transmission electron microscopy of PC-3 spheroids grown in Matrigel. mv, microvilli; sv, secretory vesicles

Analysis of PSA expression by PC-3 cells grown in Matrigel gave conflicting results. Immunohistochemistry detected PSA protein associated with PC-3 cells growing as spheroids in Matrigel but not when grown as monolayers on plastic, however the results of RT-PCR indicated that growth in Matrigel downregulated PSA expression at the transcriptional level. This apparently paradoxical effect may result from feedback repression of PSA gene expression from PSA protein retained within or around the cells in Matrigel, while PSA synthesized by PC-3 cells in monolayers may be either rapidly degraded or secreted into the culture medium. Secondly it may be that the results from immunohistochemistry are misleading due to the different cell preparations. This culture system provides an interesting model with which to study PSA expression by prostate epithelium and experiments are ongoing to further investigate the expression of PSA by PC-3 cells.

Differentiation of PC-3 cells in Matrigel is clearly not normal since they are tumour cells. They do not acquire functional E-cadherin (due to deletion of the α-catenin gene [Morton et al, 1993]) nor desmoglein, they do not polarize β1 expression and remain strong expressers of vimentin. Increased cell adhesion was apparent by transmission electron microscopy, though this was clearly not due to expression of the latter adhesion molecules. Expression of vimentin has been associated with the invasive capacity of a tumour cell and epithelial/mesenchymal conversion

 *© 2001 Cancer Research Campaign*

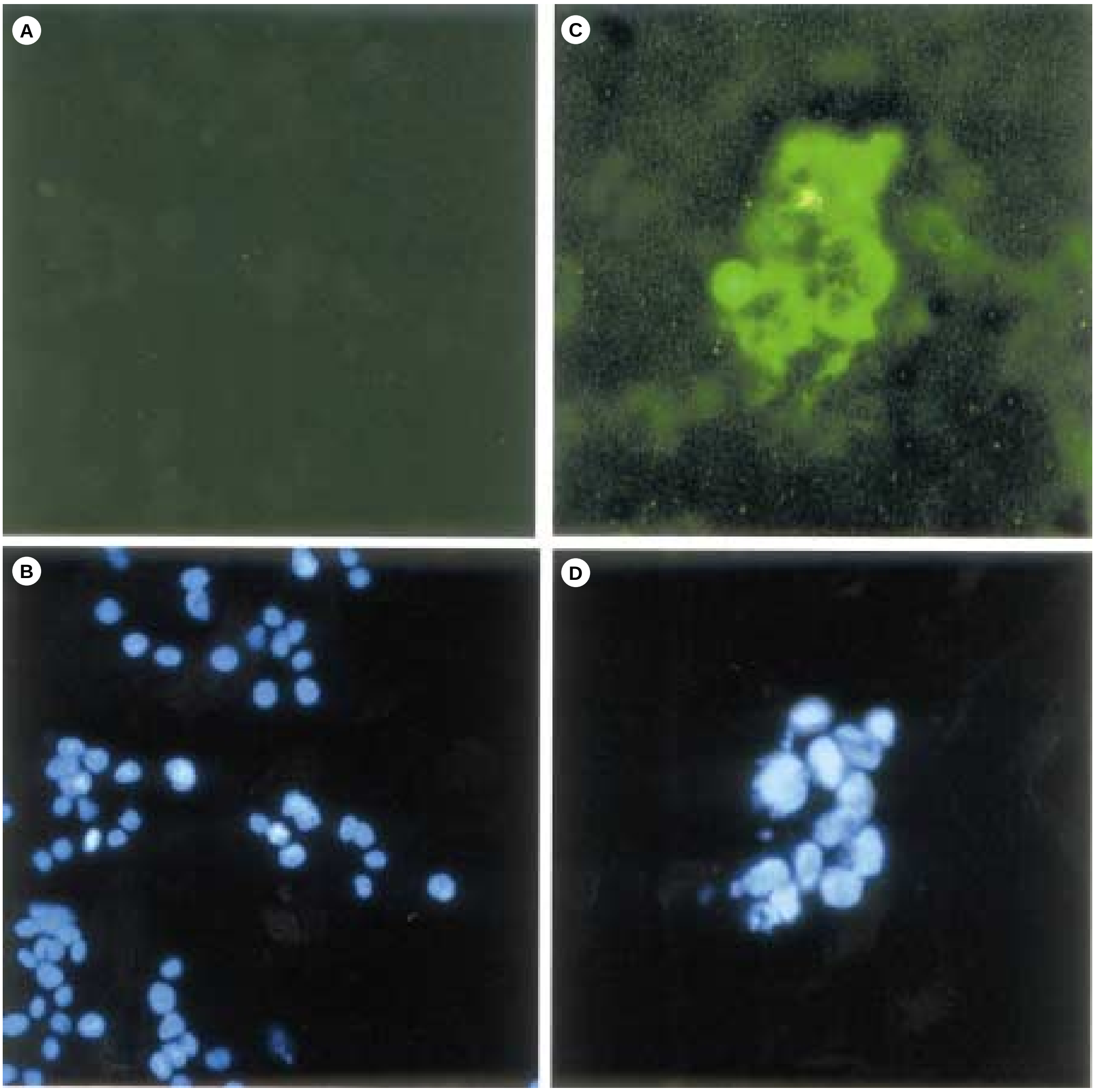

**Figure 9** Comparison of PSA expression of PC-3 cells grown in monolayer (**A and B**) and in Matrigel (**C and D**). Immunostaining for PSA is shown in (**A**) and (**C**), whilst nuclear co-staining with DAPI is shown in (**B**) and (**D**). PSA expression is cytoplasmic (**C**)

(Sommers et al, 1994; Bae et al, 1993). It is also associated with very poorly differentiated cells and possibly stem cells (Bouwens et al, 1996; Smits et al, 1996). Thus PC-3 cells are likely to represent undifferentiated basal/stem cell-like epithelia. This raises the question as to whether very invasive tumours are derived from basal or stem cells. We hope to investigate this idea further, by increasing our panel of cells to see how Matrigel influences other undifferentiated and invasive cell lines. We would also like to investigate the influence of stroma over this system since other groups have shown that stroma is required for expression of the prostate specific genes, PSA, androgen receptor and 5$\alpha$ reductase (Liu et al, 1997, Bayne et al, 1998). The altered synthesis of basement membrane as a tumour progresses may be due to alterations in the stroma, since recent work has shown that stroma derived from tumour tissue has different in vitro qualities to that derived from non-tumour tissue (Lang et al, 1999; Lang et al, 2000).

We selected Matrigel since it has been shown to induce normal epithelial cell differentiation, (Chen and Bissel, 1989; Barcellos-Hoff et al, 1989). The surprising finding that Matrigel can induce partial normal differentiation of PC-3, a metastatic cell, indicates that the altered synthesis or remodelling of basement membranes can play roles in tumour progression. In the future this may represent a suitable therapeutic target or a marker of tumour progression.

*© 2001 Cancer Research Campaign* 

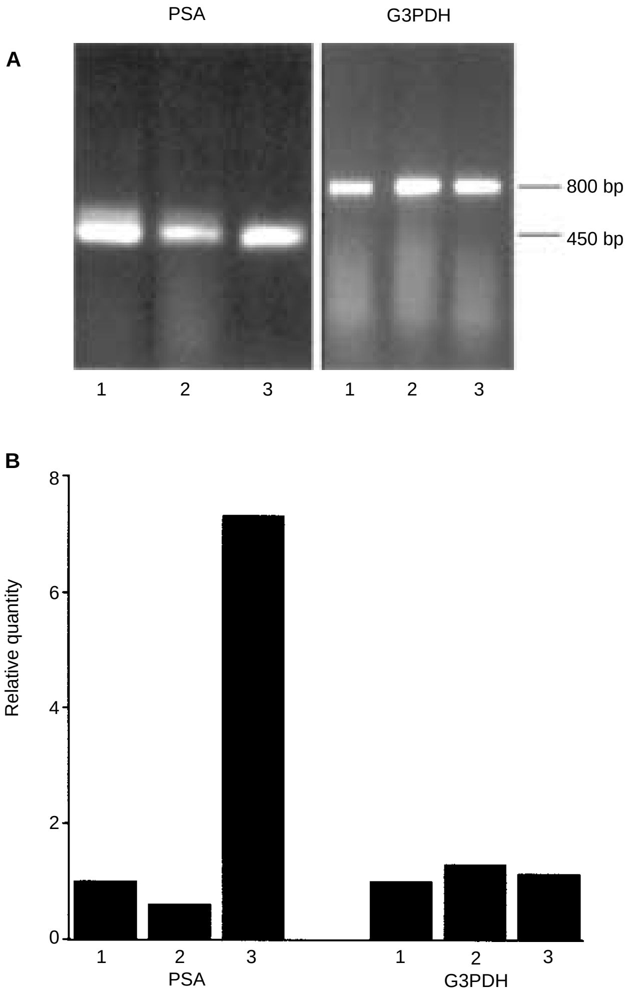

**Figure 10** RT-PCR for analysis of PSA and G3PDH expression in PC3 cells grown in monolayer culture (1) or Matrigel (2) and LNCaP cells grown in monolayer culture (3). (**A**) Equal amounts of total RNA were PCR-amplified and analysed by agarose gel electrophoresis, however to avoid overloading the gel, one tenth of the volume of RT-PCR reaction product for PSA from the LNCaP cDNA was loaded onto the gel relative to the products form PC3 cells. (**B**) The NIH image program was used to quantitate the relative band intensities from (**A**). Estimated quantities of PSA and G3PDH PCR products are shown relative to the levels in PC3 cells in monolayer culture. The value for LNCaP PSA was adjusted to account for the ratio of volumes loaded onto the gel

### ACKNOWLEDGEMENTS

Our thanks to Kath Ramsay, Katy Hyde and June Hall for their technical assistance. We are very grateful to TD Allen (CRC Paterson Institute for Cancer Research, Manchester, UK) for his expert advice on electron microscopy interpretation, and his valued interest in this work. We would also like to thank Yorkshire Cancer Research for funding this work.

### REFERENCES

Bae SN, Arand G, Azzam H, Pavasant P, Torri J, Frandsen TL and Thompson EW (1993) Molecular and cellular analysis of basement membrane invasion by human breast cancer cells in matrigel-based *in vitro* assays. *Breast Cancer Res Treat* **24**: 241–255

Barcellos-Hoff MH, Aggeler J, Ram TG and Bissel MJ (1989) Functional differentiation and alveolar morphogenesis of primary mammary cultures on reconstituted basement membrane. *Development* **105**: 223–235

Bayne CW, Donnelly F, Chapman K, Bollina P, Buck C and Habib FK (1998) A novel coculture model for benign prostatic hyperplasia expressing both isoforms of 5α-reductase. *J Clin Endocrinol Metab* **83**: 206–213

Berthon P, Cussenot O, Hopwood L, Le Duc A and Maitland NJ (1995) Functional expression of SV40 in normal human prostatic epithelial and fibroblastic cells: differentiation pattern of non-tumorigenic cell lines. *Int J Oncology* **6**: 333–343

Bouwens L and De-Blay E (1996) Islet morphogenesis and stem cell markers in rat pancreas. *J Histochem Cytochem* **44**: 947–951

Chang SS, Reuter VE, Heston WD, Bander NH, Grauer LS and Gaudin PB (1999) Five different anti-prostate-specific membrane antigen (PSMA) antibodies confirm PSMA expression in tumour-associated neovasculature. *Cancer Res* **59**: 3192–3198

Chen LH and Bissell MJ (1989) A novel regulatory mechanism for whey acidic protein gene expression. *Cell Regul* **1**: 45–54

Chomczynski P and Sacchi N (1987) Single step method of RNA isolation by acid guanidium thiocyanate-phenol-chloroform extraction. *Anal Biochem* **162**: 156–159

Cussenot O, Berthon P, Faille A, Berger R, Mowszowicz I, Teillac P, LeDuc A and Calvo F (1991) Immortalization of human adult normal prostatic epithelial cells by liposomes containing SV40. *J Urology* **143**: 881–886

Fraga CH, True LD and Kirk D (1998) Enhanced expression of the mesenchymal marker, vimentin, in hyperplastic versus normal human prostatic epithelium. *J Urology* **159**: 270–274

Knox JD, Cress AE, Clark V, Manriquez L, Affinito KS, Dalkin BL and Nagle RB (1994) Differential expression of extracellular matrix molecules and the $\alpha_6$-integrins in the normal and neoplastic prostate. *Am J Pathol* **145**: 167–174

Lang SH, Stower M and Maitland NJ (2000) In vitro modelling of epithelial and stromal interactions in non-malignant and malignant prostates. *Br J Cancer* **82**: 990–997

Lang SH, Clarke NW, George NJ and Testa NG (1999) Scatter factor influences the formation of prostate epithelial cell colonies on bone marrow stroma in vitro. *Clin Exp Metastasis* **17**: 333–340

Liu AY, True LD, LaTray L, Nelson PS, Ellis WJ, Vessella RL, Lange PH, Hood L and Van den Engh G (1997) Cell-cell interaction in prostate gene regulation and cytodifferentiation. *Proc Natl Acad Sci USA* **94**: 10705–10710

Mitchell S, Abel P, Ware M, Stamp G and Lalani EN (2000) Phenotypic and genotypic characterization of commonly used human prostatic cell lines. *BJU Int* **85**: 932–944

Morton RA, Ewing CM, Nagafuchi A, Tsukita S and Isaacs WB (1993) Reduction of E-cadherin levels and deletion of the α-catenin gene in human prostate cancer cells. *Cancer Res* **53**: 3585–3590

Murant SJ, Handley J, Stower M, Reid N, Cussenot O and Maitland NJ (1997) Co-ordinated changes in expression of cell adhesion molecules in prostate cancer. *Eur J Cancer* **33**: 263–271

Nagle RB, Hao J, Knox JD, Dalkin BL, Clark V and Cress AE (1995) Expression of hemidesmosomal and extracellular matrix proteins by normal and malignant human prostate tissue. *Am J Pathol* **146**: 1498–1507

Paradis V, Eschwège P, Loric S, Dumas F, Ba N, Benoît G, Jardin A and Bedossa P (1998) De novo expression of CD44 in prostate carcinoma is correlated with systemic dissemination of prostate cancer. *J Clin Pathology* **51**: 798–802

Pisters LL, Troncoso P, Zhau HE, Li W, von-Eschenbach AC and Chung LW (1995) c-met proto-oncogene expression in benign and malignant human prostate tissues. *J Urology* **154**: 293–298

Robinson EJ, Neal DE and Collins AT (1998) Basal cells are progenitors of luminal cells in primary cultures of differentiating human prostatic epithelium. *Prostate* **37**: 149–160

Sherwood ER, Theyer G, Steiner G, Berg LA, Kozlowski JM and Lee C (1991) Differential expression of specific cytokeratin polypeptides in the basal and luminal epithelia of the human prostate. *Prostate* **18**: 303–314

Smits A, van-Grieken D, Hartman M, Lendahl U, Funa K and Nister M (1996) Coexpression of platelet-derived growth factor alpha and beta receptors on medulloblastomas and other primitive neuroectodermal tumors is consistent with an immature stem cell and neuronal derivation. *Lab Invest* **74**: 188–198

Smola H, Stark HJ, Thiekötter G, Mirancea N, Krieg T and Fusenig NE (1998) Dynamics of basement membrane formation by keratinocyte-fibroblast interactions in organotypic skin culture. *Exp Cell Res* **239**: 399–410

Sommers CL, Byers SW, Thompson EW, Torri JA and Gelmann EP (1994) Differentiation state and invasiveness of human breast cancer cell lines. *Breast Cancer Res Treat* **31**: 325–335

Streuli CH and Bissel MJ (1990) Expression of extracellular matrix components is regulated by substratum. *J Cell Biology* **110**: 1405–1415

*© 2001 Cancer Research Campaign*

Timms BG, Lee CW, Aumüller G and Seitz J (1995) Instructive induction of prostate growth and differentiation by a defined urogenital sinus mesenchyme. *Microscopy Res Technique* **30**: 319–332
Weaver VM, Fischer AH, Peterson OW and Bissell MJ (1996) The importance of the microenvironment in breast cancer progression: Recapitulation of mammary tumorigenesis using a unique human mammary epithelial cell model and a three-dimensional culture assay. *Biochem Cell Biol* **74**: 833–851
Webber MM, Bello D, Kleinman HK and Hoffman MP (1997) Acinar differentiation by non-malignant immortalized human prostatic epithelial cells and its loss by malignant cells. *Carcinogenesis* **18**: 1225–1231

*© 2001 Cancer Research Campaign* 
